# Creating the ideal patient experience

**Published:** 2016

**Authors:** Th.V Purcărea

**Affiliations:** * Romanian-American University, Bucharest, Romania

**Keywords:** patient experience, healthcare services marketing, patient involvement and knowledge, digital transformation

## Abstract

Healthcare industry continues to evolve under conditions of intense competition in approaching health prevention, protection, and promotion. Therefore, healthcare providers are challenged to always ensure better patient experience, winning patients’ satisfaction, and loyalty and remain competitive on today’s healthcare market. Healthcare markets bring together professionals and their patients into real collaborative relationships, which empower patients to contribute to the healthcare improvement. Within this competitive landscape, which is also characterized by digital health tools boosting patients’ awareness and controlling their own health, medical providers need to be perceived as skilled and trustworthy in relying on patients’ needs, expectations, and sacrifices are required in order to obtain the promised benefits. Moreover, while constantly providing a holistic assessment of the healthcare services’ and experience attributes, acting on feedback and reaching healthcare service excellence, providing a better understanding of all the touch points with their patients and improving the quality and consistency of all these touch points, all these are achieved by employees, who are truly connected to the healthcare business.

Today, patients are systematically becoming aware of the diversity of their choices, being increasingly involved in making better healthcare choices, and, so, more and more innovative products are introduced, targeting new patient segments. Findings from the last three years have shown that patients may achieve better outcomes due to the stakeholders’ commitment to innovation within the context of the big-data revolution, by building new values.

## Reframing the core values for patient experience nowadays

On November 10, 2015, Jason A. Wolf, PhD, President of the Beryl Institute (the global community of practice dedicated to the improvement of patient experience through collaboration and shared knowledge) pledged for reframing the core values of the patient experience [**1**]. According to the Beryl Institute’s definition (2010), patient experience (PX) is “the sum of all interactions, shaped by an organization’s culture, that influence patient perceptions, across the continuum of care”. Four years later, the above mentioned president and other three distinguished colleagues highlighted the real need for a clear, comprehensive and shared definition of PX, as an important indicator (from the performance and quality of healthcare perspectives, beyond examining the provision of excellent clinical care), which is more important than satisfaction itself, as it engages patients in the care process [**2**].

In 2011, during the Patient Experience Network Conference, Joan Saddler, the National Director of Patient and Public Affairs, from the UK Department of Health [**3**], underlined the difference between PX, patient satisfaction, patient-reported outcome, and patient-defined outcome and reminded the seven key factors for achieving patient-centered care at the organizational level (Leadership at CEO and Board level; Strategic vision; Involvement of patients and families; Supportive work environment for staff; Systematic measurement and feedback; Quality of the built environment; Supportive technology), which were also identified by Dale Shaller’s report (US, 2007), and showed that PX (also related to productivity and efficiency) is closely related to and influences clinical effectiveness and safety (the “quality triangle”), keeping in mind two key issues, the importance of capturing moments at the right time in the patient journey (gathering the necessary feedback) and the determination of the main gaps of the patient survey programs. Moreover, the NHS (National Health System, UK) Outcomes framework (O/F, published on 20 December 2010 following extensive consultation) reflects the importance of PX, domain 4 of the O/F covering PX.

Patient experience is seen as a central outcome for the NHS [**4**]. A special attention is given to the different approaches of measuring patient and career experiences in health services (while collecting both detailed descriptive feedback and numerical data, weighing up the importance of depth versus generalizability, or gaining a mixture of both), and to what needs they should be measured [**5**]. Dr. Debra de Silva from “The Evidence Centre” of “The Health Foundation” also pointed out (while reviewing the empirical research about this relevant topic of measuring PX) that PX (considered in terms of the determinants of experience, the components of experience and/or the outcomes of experience; overall perceived quality, accessibility, humanization and patient involvement, as indicators of PX) is the only indicator of the healthcare quality, and it is essential to have a broader understanding of the service quality from the patients’ viewpoint by using information on both patient experience (the collection of patient-reported experience measures/PREMs) and outcomes (the collection of patient-reported outcomes/PROMs, a mandatory practice in the NHS for elective procedures), by concentrating mainly on using feedback effectively (not only on gathering it). Gathering feedback is the central challenge of teams wishing to measure PX. 

Today we are in a new phase of convergence (patients being enabled by technology) which is increased by connectivity. Digitization of our lifestyles is becoming a norm; virtualization disrupting power relationships between healthcare companies, their employees, and patients. On February 2, 2016, Jason A. Wolf, PhD, President of the The Beryl Institute [**6**] talked about the “social proximity” which is driven by connectivity and experience excellence, at the same time being defined by how much we are willing to share in the new healthcare environment.

There is no doubt that digital technology can transform the way healthcare is delivered and compensated today (the way we communicate is already transformed). For instance, the electronic health records (EHRs) create a considerable flow of information within a digital health care infrastructure, encompassing and leveraging digital progress [**7**]. Seven years ago, the Nation’s first substantial commitment of Federal resources to support the widespread adoption of EHRsin USA, was represented by the Health Information Technology for Economic and Clinical Health (HITECH) Act, as a component of the American Recovery and Reinvestment Act of 2009. In real-time, patient-centered records make information available instantly and securely to authorized users (all clinicians involved in a patient’s care). EHRs provide a workflow to providers, containing the whole patient’s medical history and other relevant information, which allows access to evidence-based tools[**8**]. In the same vein, a spontaneous online debate from April 2016 [**9**] on the impact of EHRs and the physician-patient relationship, took into account some concerns regarding the role of EHRs in caring for the patient (beyond improving the business of medicine), the existence of different EHR systems that are not interconnected (hopefully, these will converge into just a few systems that will become standardized), and the importance of what patient-centered means in a technological world. 

It is also worth recalling within this context a conclusion of a significant synthesis of a published qualitative research [**10**] concerning patient safety in primary care (research having as goal the buildingof a conceptual model), that patient safety (one of the components of the “quality triangle”, alongside PX and clinical effectiveness as shown above) can be compromised by the electronic systems when they override the opportunities of face-to-face communication between patients and the health care staff. 

## Innovation in the “Age of the Patient” and the initiation of a real patient experience management

According to McKinsey’s representatives [**11**],when it comes to innovation, no formula has been reached for success, but it is important to consider the eight components of innovation: aspire, choice, discover, evolve (the first four elements help in settingand prioritizing the terms and conditions under which innovation is more likely to thrive), accelerate, scale, extend, and mobilize (the last four deal with how to deliver and organize the repeated innovation over time). Paraphrasing Forrester [**12**], the author’s opinion is that in the “Age of the Patient” there are four priority areas in which patient-centered healthcare providers have to invest, namely, real-time actionable data sharing, contextualized patient experiences across touch points, sales efforts tied to buyers’ processes, content-led marketing and patient interactions, and the followingof four market imperatives: transforming the patient experience, embracing the mobile mind shift, becoming a digital disruptor and turning big data into healthcare business insight [**13**].

On the other hand, taking into account the opinion of Bob Thompson[**14**], CEO of CustomerThink Corp. (an independent research and publishing firm focused on customer-centric business management) and Founder/Editor-in-Chief of CustomerThink.com (the world’s largest community dedicated to customer-centric business), it should be added that, in order to have a real “Patient Experience Management” (PXM) initiative it is advisable to follow some steps: helping healthcare providers better plan their actions, preparing and managing their healthcare business, knowing the healthcare brand purpose and promise, taking an outside-in perspective (considering patient research and feedback management – the “Voice of Patient”/VoP), developing “Patient Journey Maps” (comparing the existing and desired experiences as a good foundation for a PX plan), empowering healthcare provider employees (so as to be prepared and motivated to deliver the right PX), creating a healthcare business case by connecting PX improvements (what patients want) to healthcare business outcomes (what healthcare providers CEOs demand). Further, the “Patient Journey Maps” could be useful for healthcare providers to adopt the “Modern Customer Service: Mapping the Journey Ahead” approach designed by Oracle, [**15**]. In other words, healthcare providers must embrace the modernization as an “evolution” (not like a “revolution”), seeing and serving patients across all engagement channels (including traditional and new channels), better understanding that an effective communication is the key to success (being at the root of most failures when it is missing) and service drives PX (the last one being about establishing and maintaining a strong relationship with customers, while patient service and support –3/4 of interactions with patients – is about the delivery of the healthcare brand promise which builds trusted relationships). Last, but not least, it is important to take into account the “PX Continuum Journey”, as in awareness, trust, commitment, honeymoon, relationship, partnership, and advocate [**16**].

In the beginning of February 2016, while interviewing Professor Bettina Borisch [**17**], a trained medical doctor and histopathologist, at the World Federation of Public Health Associations (WFPHA), the importance of improving communicationwas highlighted with the help of the health community which must accept that there are two-ways discussions: between the patient (who no longer has a passive role, wishing to discuss the acquired knowledge) and the doctor. Professor Borisch also pointed out the need of a better (from a financial perspective) prevention support and general wellbeing (compared to care and treatment).

In a nutshell, there is an accordance between the ideas expressed above and the ones found in the “Guiding principles” underlined by the Beryl Institute [**18**] such as: engage all voices in driving comprehensively, systemic and lasting solutions, look beyond clinical experience of care to all interactions and touch points, focus on alignment across all segments of the continuum and the spaces in between, encompass both a focus on healing and a commitment to well-being.

It is no coincidence that several valuable ideasabout the creation of the ideal patient care experience were reiterated recently [**19**], and were materialized underhealthcare experiences, which reflect situations of people who remember with great intensity, having an ideal quality.Healthcare must be safe, effective, patient-centered, timely, efficient, and equitable; patient and family centered care (PFCC) is a philosophy, a process and a practice in order to transform the care experience and improve quality and safety. The PFCC key principles are dignity and respect, information sharing, participation, and collaboration (mshpm.org, 2016). Within this framework, it is also worth remembering some other valuable ideas coming from the Center for Innovation, at the Mayo Clinic: Patients are experts in their diagnosis, and their expertise is worth something, serving as acknowledgement! Let’s turn patients’ ideas, thoughts, and insights from their social identitiesinto actionable change in the physical world of healthcare! Healthcare will be truly transformed by forming teams of patient experts and healthcare industry “insiders” to take on projects! [**20**]

## Redesigning healthcare delivery systems

McKinsey’s representatives [**21**] pointed out that change efforts are hard work and thus, implementation is critical to the overall transformation success, shifting mind-sets and behaviors. The change in behaviors is a science of organizational transformation (building on strengths, treating design as a science, focusing on the “what” and the “how”, involving multiple stakeholders, etc.). At the same time, there is a real need to combine the technologies (digital, social, and big data and analytics) to create a competitive advantage [**22**]. It seems that McKinsey 7-S model (Hard S’s: strategy, structure, systems; Soft S’s: style, staff, skills, superordinate goals/ shared goals) still works [**23**].

According to an authorized opinion – about healthcare models – expressed in 2014 [**24**], about the changes in mindsets and behaviors (none of them can be mandated or dictated), there is an urge need to occur at all levels of the organization, starting with the front-line physicians, clinical team, and physician leadership. On the other hand, ChenMed, a primary care-led physician group, considers that there are four key drivers of success in integrated care, namely, focus on the patient relationship (entire team owns the relationship; relationship evolves over time; >85% of the touch-points), physician decision-making (selection and culture; decision support at point of care; positive incentives – the "tuned" patient panel), convenience matters (redesigned system of on-site physician drug dispensing dramatically improves adherence; on-site behavioral health model coordination), and communication (coordination of care; specialist – PCP communication in person; team conference; 3 times a week review of patient care by the physician group; transparent review of outcomes with all physicians). Moreover, according to another authorized opinion [**25**] expressed in 2015 (looking at accelerating patient experience improvement in ambulatory care) it was stated that creating the ideal patient and family experience suggests setting the service expectation, the ideal visit, continuous improvement, and making an impact. Emory Clinic Healthcare approach presented at Beryl Patient Experience Conference on April 9, 2015included: Service improvement (service performance consultants, patient advocacy, service training), PX (volunteers, guest services, access special constituent patients), patient feedback (Press Ganey [**26**], STARS patient complaints& grievances, secret shopping), Patient Satisfaction Acceleration Team (PSAT - formed 2 years ago; has met at every two weeks with all decision makers in attendance; filtered out the "noise" – 100,000 completed surveys necessary to improve: ease of scheduling, ease of getting the clinic on the phone, wait time at clinic, and sensitivity to patient’s needs).

Another interesting example of a good PX approach is “Creating the Exceptional Patient Experience in One Academic Health System [**27**] which was launched in February 2008 by the University of Utah Health Care system and was called the Exceptional Patient Experience (EPE), having the following slogan “Medical care can only be truly great if the patient thinks it is”. This project began as a patient satisfaction initiative, but has evolved into a model for cultural transformation (physician engagement, value-based employment practices, enhanced professionalism and communication, reduced variability in performance, and improved alignment of the mission and vision across hospital and faculty group practice teams), becoming the cornerstone for other project initiatives (focused on quality and safety, patient-reported outcomes, and cost reduction).

Today, healthcare is considered an exceedingly complex ecosystem of competing priorities and legacy systems, and an ecosystem under the pressure of radically increased patients’ expectations of experience, transparency and access to healthcare market [**28**]. Within this challenging context, Michael Hinshaw (President/CEO of McorpCX, believes that a customer experience company helping companies radically improve business performance by transforming how they interact with their customers, recently gave five examples of redesigned delivery systems of better getting, serving and keeping these new smart and empowered patients: “Tele-medicine” (talking with a real doctor, on your schedule, where you are); “Doc-in-a-robot-shaped-box” (the physician can see the patient without being with him, but providing continuous monitoring); “Concierge Medicine” for the masses (same-day appointments, online scheduling); “Trust but verify” (second medical opinion for complicated diseases and diagnoses, improving PX and confidence in treatment choices); “Your health data, and your health” (Big Data becoming a tool for the improvement of healthcare; personalized health applications enabling the storing and management of personal health data in empowering ways).

The author adds that we are living interesting times, of revolution in healthcare (brought about by the use of technology), providing the opportunity to help patients create “positive habits” that will improve their health, as recommended recently, for example, by United Healthcare [**29**], a major insurer which replaced its previous customer wellness program with “Rally” (a more comprehensive one). Therefore, it is time to rally, calling together for the common purpose of creating the ideal PX.

## Implementing healthcare marketing and considering the priorities in ensuring the ideal patient experience 

Paraphrasing Peter Drucker [**30**] (while illustrating the marketing era/concept in 1954), the author states that what the patient considers “value” is decisive, because it determines what a healthcare business is, what it produces, and whether it will prosper. Considering this decisive “value” for the patient, the author adds, by paraphrasing C. Lovelock and J. Wirtz [**31**], that it is important to implement healthcare services marketing accordingly, by managing relationships and building loyalty, considering patient feedback and service recovery, improving healthcare service quality and productivity, organizing for healthcare service leadership), understanding the challenge of building relationships with patients, including preference, liking, future intentions, etc., and being also able to identify the reasons why patients defect and then take a corrective action.

There is also a growing recognition of the significant importance of patient involvement and knowledge. For example, hospitals – in the light of patient empowerment and channel fragmentation – struggle to target patients in their new role as knowledgeable consumers [**32**]. Among others, it is considered that market differentiation via traditional marketing mix variables such as price, is hardly achievable in healthcare, and that superior communication grounded on sound positioning is a more promising lever for competitive advantage. As a main care coordination pivot, hospitals need a better understanding of integrated marketing communications, by using positioning as the proper strategy while implementing these communications, tailoring the message to patient segments accordingly.

Also, it is important to better understand the patient-perceived hospital service quality [**33**] by making the difference between the nature of technical quality (which encompasses credence attributes) and the functional quality (which is based on experience properties of healthcare service), patients being well qualified to judge the last one. It is well known that there is a widespread implementation of the “Consumer Assessment of Healthcare Providers & Systems Hospital Survey” (HCAHPS) instrument (focused on perceived functional quality component).

There is no doubt within this general context that healthcare marketers are convinced that patients are more and more eager to access information that empowers them, and person-to-person relationship in healthcare is becoming increasingly essential. In June 2015, a healthcare marketing vice-president in Southern California and a marketing instructor at four universities [**34**]talked about opportunities and lessons for a healthy relationship, within the framework of the so-called phenomenon of “cyberchondria”. There are already many tools answering new expectations by providing insightful and usually real-time data, such as: smart watches (capturing user heart and pulse rates, fitness activities, etc.), in-home lab tests, exercise trackers, portable ECGs, etc. He suggested seven lessons to address healthcare marketers, as it follows: “don’t tussle for data, share it…; explain what the data means…; if you don’t share data, someone else will…; customers who want data are active in online communities…; not everyone is on the same path…; ensure that everyone in your organization respects customer data…; data is a tool to facilitate the age-old desire to establish a relationship…”

Findings from a UK study [**35**] showed that patients used elements of organizational culture as resources to help them collaborate with healthcare professionals, and when patients (beyond simply providing their views as receivers of care) find such ways to support their involvement in the healthcare improvement, they can influence healthcare in unexpected ways. However, this engagement in healthcare improvement (beyond simply providing their views as receivers of care) defines a dynamic interplay between psychological and sociocultural processes, essential for effective patient participation in the process of improving the healthcare provider organizational cultures.

Healthcare providers in USA are paying a particular attention to how each patient perceives the care they received, in the specific Affordable Care Act (ACA) environment, PX scoringbeing more important than ever [**36**]. It is well-known that the partnership (in order to improve clinical and business outcomes by assessing the total PX) between the majority of healthcare providers and the above mentioned Press Ganey (Press Ganey Score functioning comparably to a B2B Net Promoter Score for more than 30 years) ratings are based on the healthcare provider’s ability to create and sustain a high-performance environment that ultimately improves PX. In the ACA (Affordable Care Act) environment, patient experience scoring is also important. The Vice President and Chief Marketing Officer at Qmatic Group (a world leader creating better customer journeys, and remarkable customer experiences, who has spent more than three decades helping healthcare providers to create the best possible patient experience by gathering feedback data), recommended some priorities, as for instance, a simplified appointment scheduling; creating a friendly environment; reducing perceived waiting times; lessening confusion; surveillanceof customers in real time; following up with the patient (top quality care involving ongoing communication). The Qmatic Group’s representative concluded that in order to create the care environments that patients appreciate most, it is more important than ever to remain connected, aware, and agile in today’s complex healthcare compliance atmosphere. 

## Conclusions

On February 2, 2016, Jason A. Wolf, PhD, President at The Beryl Institute suggested that the most important thing is to bring silence to our work in patient experience, while striving to achieve in caring for one another. Starting from this profound humanist message, the author added a “to do list” (inspired by the challenging competitive business environment) for healthcare providers, such as: put people (patients and employees) at the center of everything the healthcare provider does; create healthcare business objectives to be achieved within a specific time frame and with specific resources; examine healthcare provider’s employee experience in parallel with the patient experience; optimize patient experience programs to strive for enhanced levels of patient brand passion, bonding, and advocacy as well as employee ambassadorship; sustain patient experience initiatives by leadership discipline and consistency; build a PX framework, including a patient decision journey map.

## References

[1] Wolf JA (2015). Reframing the Core Values for Patient Experience. http://blog.theberylinstitute.org/.

[2] Wolf JA, Niederhauser V, Marshburn D, LaVela S (2014). Defining Patient Experience. Patient Experience Journal.

[3] Saddler J (2011). Patient Experience Network Conference. NHS.

[4] (2010). Equity and excellence: Liberating the NHS. Presented to Parliament by the Secretary of State for Health by Command of Her Majesty. https://www.gov.uk/government/uploads/system/uploads/attachment_data/file/213823/dh_117794.pdf.

[5] De Silva D (2013). Measuring patient experience. The Health Foundation, Evidence scan. The Evidence Centre.

[6] Wolf AJ (2016). Silence: The Invisible Tool for Patient Experience Excellenc. http://blog.theberylinstitute.org/.

[7] Levingston SA (2012). Opportunities in physician electronic health records: A road map for vendors. Bloomberg Government, cited in Benefits of Electronic Health Records (EHRs). https://www.healthit.gov/providers-professionals/benefits-electronic-health-records-ehrs.

[8] Electronic Health Records: The Basics. https://www.healthit.gov/providers-professionals/frequently-asked-questions/334#id2.

[9] Branche LC (2016). EHRs are ruining the physician-patient relationship. http://medicaleconomics.modernmedicine.com/medical-economics/news/ehrs-are-ruining-physician-patient-relationship.

[10] Daker-White G (2015). Blame the Patient, Blame the Doctor or Blame the System?. A Meta-Synthesis of Qualitative Studies of Patient Safety in Primary Care. EndocrinolMetabClin North Am.

[11] De Jong M, Marston N, Roth E (2015). The eight essentials of innovation. McKinsey Quarterly. Arq Bras EndocrinolMetabol.

[12] http://solutions.forrester.com/ageofthecustomer/landing-307PF-278755.html.

[13] McQuivey J (2013). The secrets of digital disruption. http://www.computerweekly.com/opinion/Forrester-The-secrets-of-digital-disruption.

[14] Thompson B (2016). Top 5 signs you’re doing a REAL Customer Experience Management (CXM) initiative. Minerva Chir.

[15] (2015). Modern Customer Service. Mapping The Journey Ahead.

[16] Buehrens C (2016). 10 Kick-“As” Ways to Use Customer Experience Journey Maps. Rutgers Center for Innovation Education. SurgEndosc.

[17] (2016). Thinking global for better public health. http://www.internationalinnovation.com/thinking-global-better-public-health/.

[18] Wolf AG (2015). Taking a Stand for Patient Experience Excellence. http://blog.theberylinstitute.org/.

[19] Creating the Ideal Patient Care Experience Michigan Society for Healthcare. Planning and Marketing.

[20] (2016). Spring Conference.

[21] DeLaO A (2015). What are these coins? The my ideal patient experience project. http://blog.centerforinnovation.mayo.edu/2015/12/16/what-are-these-coins-the-my-ideal-patient-experience-project/.

[22] Basford T, Schaninger B, Viruleg E (2015). The science of organizational transformations. Survey McKinsey.

[23] Bughin J, Chui M, Harrysson M (2016). How social tools can reshape the organization. Survey McKinsey Global Institute. http://www.mckinsey.com/business-functions/mckinsey-digital/our-insights/how-social-tools-can-reshape-the-organization?cid=other-eml-alt-mgi-mgi-oth-1605.

[24] Basford T, Schaninger B (2016). The four building blocks of change. McKinsey Quarterly.

[25] Alvarez J (2014). ChenMed Care Model: Creating Change and Transformation in General Practice. J Med Assoc Thai.

[26] Dubovsky A (2015). Accelerating patient experience improvement in ambulatory care. Emory Clinic. Beryl Patient Experience Conference.

[27] http://www.pressganey.com/about.

[28] Lee VS, Miller T, Daniels C, Paine M, Gresh B, Betz AL (2016). Creating the Exceptional Patient Experience in One Academic Health System. Academic Medicine.

[29] Hinshaw M (2016). Patient Experience Innovation: 5 Disruptive Examples. http://customerthink.com/patient-experience-innovation-5-disruptive-examples/.

[30] Bobnak P (2016). United Healthcare Email Makes Getting Healthier a Game. http://www.targetmarketingmag.com/article/united-healthcare-email/#utm_source=today-%40-target-marketing&utm_medium=newsletter&utm_campaign=2016-05-04&utm_content=united+healthcare+email+makes+getting+healthier+a+game-.

[31] Drucker P (2006). The Practice of Management 1954.

[32] Lovelock C, Wirtz J (2007). Services Marketing: People, Technology.

[33] Fischer S (2014). Hospital Positioning and Integrated Hospital Marketing Communications: State-of-the-Art Review, Conceptual Framework, and Research Agenda. Journal of Nonprofit & Public Sector Marketing.

[34] Westbrook WK, Babakus E, Cohen Grant C (2014). Measuring Patient-Perceived Hospital Service Quality: Validity and Managerial Usefulness of HCAHPS Scales. Health Marketing Quarterly.

[35] Solomon S (2015). Seven Opportunities and Lessons for a Healthy Relationship With Data. http://www.marketingprofs.com/articles/2015/27930/seven-opportunities-and-lessons-for-a-healthy-relationship-with-data#ixzz3ejoIEWh.

[36] Renedo A, Marston CA, Spyridonidis D, Barlow J (2015). Patient and Public Involvement in Healthcare Quality Improvement: How organizations can help patients and professionals to collaborate. Public Management Review.

[37] Husmark S (2016). 6 Steps for Creating the Ideal Patient Experience The Affordable Care. http://customerthink.com/6-steps-for-creating-the-ideal-patient-experience/?utm_source=subscriber.

